# Effect of the Thumb Orientation and Actuation on the Functionality and Performance of Affordable Prosthetic Hands: Obtaining Design Criteria

**DOI:** 10.3390/biomimetics7040233

**Published:** 2022-12-11

**Authors:** Javier Andrés-Esperanza, Jose L. Iserte-Vilar, Immaculada Llop-Harillo, Antonio Pérez-González

**Affiliations:** Department of Mechanical Engineering and Construction, Universitat Jaume I, 12071 Castellón, Spain

**Keywords:** thumb, hand, prosthesis, underactuation, 3D printing, benchmarking

## Abstract

The advent of 3D printing technologies has enabled the development of low-cost prosthetic underactuated hands, with cables working as tendons for flexion. Despite the particular relevance to human grasp, its conception in prosthetics is based on vague intuitions of the designers due to the lack of studies on its relevance to the functionality and performance of the device. In this work, some criteria for designers are provided regarding the carpometacarpal joint of the thumb in these devices. To this end, we studied four prosthetic hands of similar characteristics with the motion of abduction/adduction of the thumb resolved in three different ways: fixed at a certain abduction, coupled with the motion of flexion/extension, and actuated independently of the flexion/extension. The functionality and performance of the hands were assessed for the basic grasps using the Anthropomorphic Hand Assessment Protocol (AHAP) and a reduced version of the Southampton Hand Assessment Procedure (SHAP). As a general rule, it seems desirable that thumb adduction/abduction is performed independently of flexion/extension, although this adds one degree of control. If having this additional degree of control is beyond debate, coupled flexion/extension and adduction/abduction should be avoided in favour of the thumb having a fixed slight palmar abduction.

## 1. Introduction

Mechanical replication of a biological hand in general, and its thumb in particular, is one of the current challenges in the prosthetic and robotic fields, but it is far beyond the reach of today’s technologies. However, the presence of the thumb in the hand is highly required. Anatomists highlight that the hand, without a thumb, loses most of its capabilities, thus being “nothing but an animated spatula and, at best, a pair of forceps whose points do not meet properly” [1]. 

**Figure 1:**
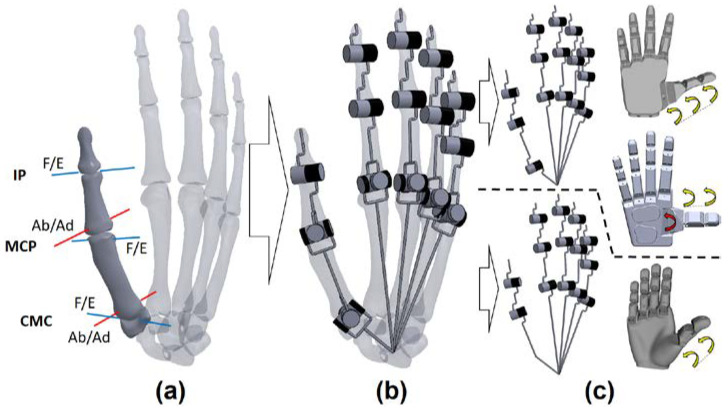
(**a**) Joints of the human thumb; (**b**) idealised kinematic chain (KC) for artificial hands; (**c**) simplified KCs for the Dextrus, IMMA, and Limbitless hands, as shown from top to bottom. Yellow arrows: several DOF actuated by the same actuator (underactuation); red arrows: DOF actuated by one independent actuator.

Theorising about the kinematic chain of the thumb is cumbersome. The classical mechanical models for the joints simplify their actual motion, which results from the flexibility and the complex sliding/rolling motion of the bone heads. Figure 1a shows the kinematic model of the thumb with 5 degrees of freedom (DOF) which best approximates the anatomical movements, where the joints with 2 DOF have non-orthogonal and non-intersecting axes [2]. The interphalangeal (IP) is the simplest joint and is considered a hinge joint (1 DOF). The carpometacarpal (CMC) joint, also known as trapeziometacarpal (TMC), is of saddle shape type (2 DOF, Figure 1b) and best approximated by a joint of hyperboloid geometry. The MCP joint has a condyloid type (2 DOF) with an important lateral and rotational (twist) DOF [3]. All 5 DOF of the human thumb are involved in achieving opposition: three DOF are necessary to make one point of the thumb’s fingertip meet the position of another point inside the reachable space of the thumb’s kinematic chain, and two additional DOF are necessary for the planes of the finger-pulps to match [4,5]. 

**Figure 2:**
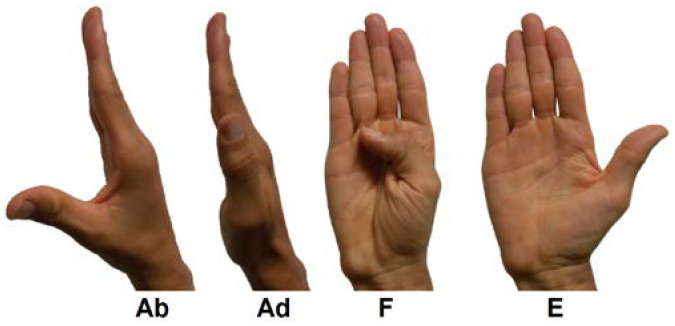
Abduction/Adduction (Ab/Ad) and Flexion/Extension (F/E) of the thumb.

Beyond this kinematic configuration, the vastly sophisticated neuromuscular system of the hand gives the human brain an enormous ability for manipulation. The joints of the human thumb are actuated via muscles and tendons. The thumb muscles are nine skeletal muscles, five located within the hand (intrinsic) and four with muscle bellies in the forearm (extrinsic hand muscles). Muscles never work independently, and even the simplest motion comes from the coordinated and averaged action of several of them [6]. As stated before, the opposition motion represents the main functionality of the thumb and allows all configurations between the flat hand and the opposition for pinch and power grasps. The pure movements of the thumb’s metacarpal through the CMC joint are those of abduction/adduction (Ab/Ad) and flexion/extension (F/E) [4]; see Figure 1a and Figure 2. Specifically, combined F and Ab produce CMC opposition, and E and Ad produce CMC reposition [7].

Nowadays, the popularisation of the FDM (fused deposition modelling) 3D-printing technology has enhanced non-profit initiatives [8,9] seeking to provide affordable prosthetic hands (normally, less than $500) for the 2.4 million upper-limb amputees living in low and medium-income countries (LMICs) [10,11]. Under the Do It Yourself premise [12,13], the altruistic designers feed Computer-Aided Design (CAD) repositories (such as www.instructables.com (accessed on 16 September 2021) and www.thingiverse.com (accessed on 16 September 2021)) from where anyone can freely download a ready-to-print prosthetic hand. 

From a mechatronics perspective, Controzzi et al. [14] identified six key issues to be considered in the design of such prosthetic hands, namely: (a) kinematic architecture, (b) actuation principle, (c) actuation transmission, (d) sensors, (e) materials, and (f) manufacturing method. The existence of a large number of affordable devices has prompted some reviews of the state of the art that can be contrasted with these key issues. Phillips et al. [10] examined 18 prostheses with a focused overview of the materials and actuation for each device and a discussion of their limitations. Ten Kate et al. [11] reviewed 46 current FDM upper limb prostheses, giving quantitative information about them. Burn et al. [15] and Tanaka et al. [16] looked over some of the most prevalent prostheses aimed at children. These studies make evident the predominance of underactuation (b) (i.e., having fewer degrees of control (DOC) than DOF [14]) using nylon threads running into sheaths (c) as tendons for the digits. These tendons link the motion of the joints at the digits in order to close the hand by pulling them. In the context of affordable designs, hands have no feedback from any sensor (d). The materials (e) normally used in FDM 3D-printers (f) are acrylonitrile butadiene styrene (ABS) or polylactic acid (PLA), also used in conventional orthotics [15,17]. Additionally, the use of elastic cords or thermoplastic elastomers (such as Ninjaflex^®^) in the joints may avoid the need for a digit extension system [18,19]. Regarding the underactuation (b), slightly more than half of all the prostheses in these studies were body-powered (BP), i.e., these tendons are pulled remotely by moving another part of the body as the only DOC, with all fingers bending together. Some models may include DC motors located in the palm or the forearm in order to achieve more precise grasping postures by having more DOC. All in all, prostheses designed for LMICs exhibit simplified designs. Both assembly and maintenance are very easy in these kinds of hands pulled by tendons. 

In the scope of these affordable designs, one key factor in succeeding in finding the proper distribution of the digits on the palm and especially of the thumb, i.e., kinematic architecture (a). In fact, the primitive orientation of the thumb in the hand has aroused little discussion amongst designers, yet it has crucial importance for the opposition of the thumb [2]. In affordable designs, the two DOF of the CMC joint are usually simplified to the unique DOF of a hinge joint with a permanent orientation or even suppressed (see Figure 1c). This leaves the thumbs in these affordable hands with three or two DOF, respectively. Regarding the range of motion (ROM) of these movements, designers are committed to the idea of getting a natural motion between the values documented in medical sciences (46°/−14° in F/E, and 25/−8° in Ab/Ad) [20]. They assume that the natural disparities in the ROM of the thumb among individuals do not impair manipulation skills as long as the stability of the thumb is maintained [21]. In this sense, the designer’s main aim has been to provide a sufficient opening angle for common objects [22]. Only a few authors exposed some guidelines for the design of the thumb but in the context of robotic hands, which are much more technologically complex than those in the scope of this research [21,22,23,24,25].

**Figure 3:**
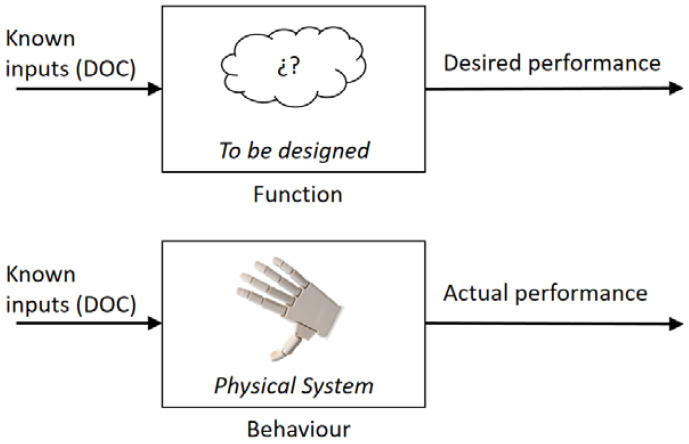
Function and behaviour.

The difficulty in putting together and maintaining small integrated mechanisms, such as those used in robotics or more expensive electric hands [26,27], explains why the designers often use simplified conceptions of the thumb for affordable hands [19]. Nevertheless, this conception attends to the designers’ intuition. None of the aforementioned studies evaluated the performance of the hand. Therefore, in order to regain the ultimate goal of providing solutions from the designer’s point of view, evaluation of the performance must condensate the two concepts of function and actual behaviour. Function and behaviour are usually taken as synonyms. However, there is a subtle difference (see Figure 3) [28]: on the one side, the function is the desired outcome from a device that may even be yet to be designed; on the other side, if the device exists, its behaviour can be ascertained. In the context at stake, behaviour is the actual response of the prosthetic hand to the control inputs, aiming to serve a specific task (at this point, desired performance). Thus, the behaviour can be measured by registering and scoring the actual response, whereas any implemented function is only a means. 

Lately, numerous high-technological hand prostheses have been confronted with the SHAP [29] with diverse outcomes [30,31,32]. It has also raised some criticisms about the applicability of the SHAP [33,34,35], which prompted a proposal for a reduced version of the procedure [35]. 

This research assessed the performance and the functionality of four 3D-printed hand prostheses by confronting them to this reduced SHAP and the AHAP [36], respectively. As the focus is on the influence of the thumb, two of the hands are different versions of the same model, only varying the orientation that the thumb has after fusing the CMC joint within the palm body. The other two hands incorporate one DOF at the CMC joint of the thumb. One of these last two hands has been developed by our group based on the other models studied here, but considering two DOC for the thumb instead of one. These assessments are intended to address the importance of the orientation and the minimum DOF of the thumb in affordable prosthetic hands and how its control should be managed in order to maximise the user experience. After presenting the four hands and the test protocols, the results will be presented for discussion. Finally, conclusions on DOF and DOC in these hands will be drawn.

## 2. Materials and Methods

### 2.1. Affordable Prosthetic Hands 

**Figure 4:**
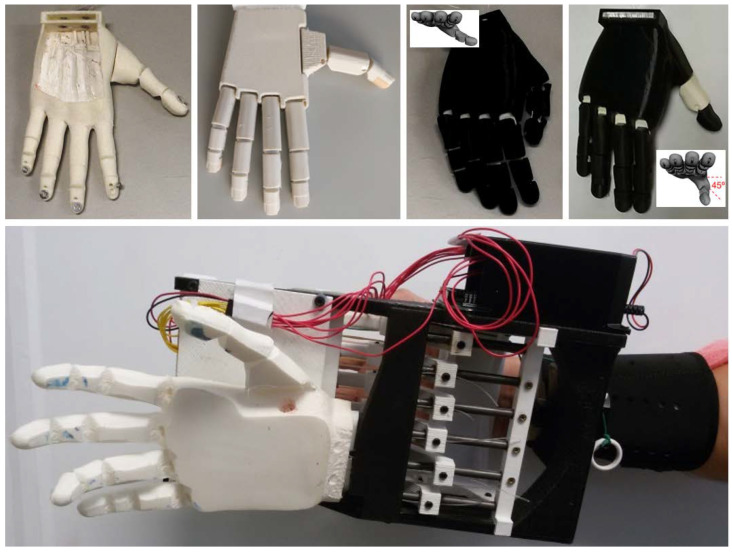
Affordable prosthetic hands, from left to right: Dextrus, IMMA, and Limbitless hands. The two versions of the Limbitless are depicted. Below is Able-Bodied Adaptor (ABA).

The four affordable prosthetic hands proposed in this research to evaluate their performance and, in particular, the influence of the thumb are Dextrus v2.0, Limbitless hand (in two versions, Lb-0 and Lb-45), and the IMMA hand (developed in our group). As these hands try to mimic the human hand, the joints of the fingers in these prosthetic devices will be named by analogy, from distal to proximal: distal interphalangeal (DIP), proximal interphalangeal (PIP), metacarpophalangeal (MCP), and CMC. Note that the thumb has only one IP joint and that not all thumbs have been designed with the same number of joints. Mimicked joints are listed in Table 1. Some details of these prostheses are (see Figure 4 and Table 1):

- Dextrus v2.0 [37]: This hand has flexible joints fully integrated within its rubberized and flexible unibody design made of Ninjaflex^®^. After printing, the nylon threads just need to be routed. There is no need for assembly, although the substitution of individual fingers is impossible if broken. Particularly, the thumb presents three DOF (IP, MCP, and CMC hinge joints) underactuated by one tendon. In the present document, we will recall this model simply as Dextrus.
- Lb-0 and Lb-45 (Limbitless) [38,39]: the original design of the Limbitless hand was developed by the University of Central Florida Armory on the basis of the wrist BP Flexy-Hand [40]. In this device, the CMC joint of the thumb is absent, the first metacarpal being fused to the palm body. This leaves only two DOF for the thumb (IP and MCP hinge joints). It is available either without palmar abduction or with a palmar abduction of 45 degrees of the thumb, see Figure 4. We will recall these designs as Lb-0 and Lb-45, respectively. Any Limbitless hand was originally intended to work with one actuator in the forearm or beyond (one DOC), thus closing fingers and thumb together. In the present study, however, this fact was unobserved, and each tendon was pulled independently for each digit, thus having five DOC.
- IMMA hand [41]: inspired in some existing affordable hands such as the Dextrus and the Limbitless, the main characteristic is that the movements of the thumb are actuated separately by means of two different tendons, thus having two DOC for the thumb: one for flexion at IP and MCP joints, and another for abduction at the CMC. Different materials based on different combinations of PLA and thermoplastic polyurethane (TPU) were used in the construction of the hand. In the present document, we will recall this model as IMMA.

The same 0.8 mm nylon thread (ultimate tensile stress of 220.5 N) was used as a tendon in each digit. All of them were manufactured using FDM: time and cost were recorded from our experience with a Colido^®^ X3045 3D printer, with Repetier-Host (www.repetier.com (accessed on 2 April 2021)) software. For each hand and prior to FDM printing, a wrist add-on with two holes was merged with the CAD model of each hand to make it easier to fasten them to the able-bodied adaptor (ABA) described in the following section.

### 2.2. Able-Bodied Adaptor (ABA)

Based on a former design by the authors [41], the ABA shown in Figure 4 was designed and 3D printed, seeking a smaller distal separation of the artificial hand to the subject’s arm [42]. It is attached to the forearm of an able-bodied subject using a Pro Cuff^®^ (www.trsprosthetics.com (accessed on 23 April 2021)) and allows controlling any of the prosthetic hands presented in the previous section by pulling each tendon with one’s own fingers. The ABA may accommodate hands with up to 6 DOC, with the sixth being conducted from the other hand. It should be noted that SHAP has proven to give equivalent scores for patients with limb loss and able-bodied subjects with the aid of an ABA [43], so most of the research in the literature has been done in this second way (see [32,44,45,46,47,48,49,50]).

Using this ABA, four able-bodied subjects participated in the experiments described below. Getting familiar with ABA is easy because it involves the natural task of pulling the thread corresponding to each finger with visual, haptic, and proprioceptive feedback. The Ethics Committee of the Universitat Jaume I approved the study, and written informed consent was obtained from all participants.

### 2.3. Methods

**Figure 5:**
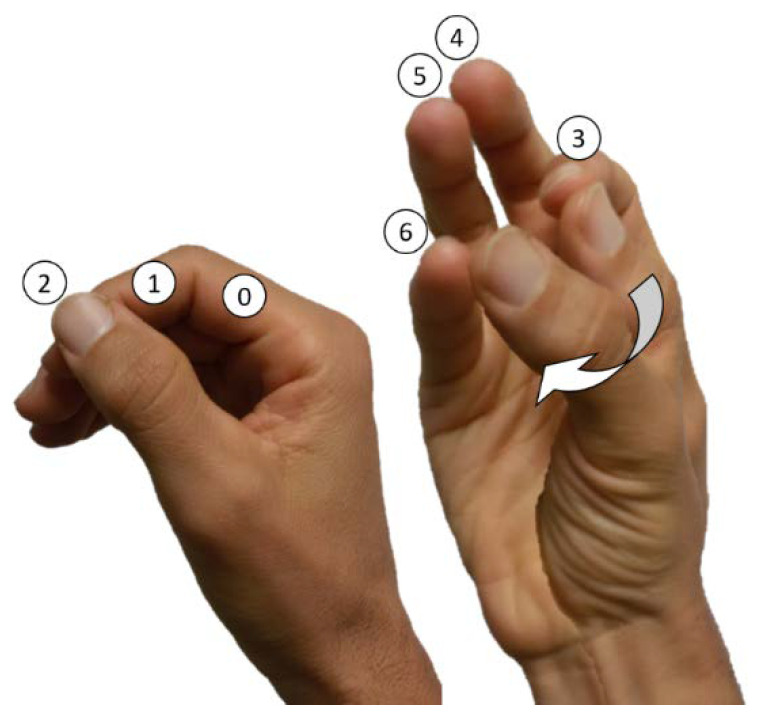
Definition of the seven points for the Kapandji opposition test in a human hand.

Traditionally, functional evaluation of the human hand has observed some postures of the hand, such as the Kapandji test [5,51], or simplified actions, such as the Jamar pinch & grasp tests [52,53]. The Kapandji test requires moving the tip of the thumb to a list of predefined locations in the own hand, having thus to adopt a set of postures that may give insight into some minimum abilities from a kinematic point of view (see Figure 5). Undoubtedly, the influence of the thumb on the results of this test is paramount. It is aimed at the human hand, e.g., in evaluating rehabilitation processes, yet we must be aware that we are not evaluating a human hand but a prosthetic device. 

That said, prosthesis users clearly perceive their prostheses as assistive devices and the type of activities for which they are intended (see [33]). In this research, we focused on two sets of tasks to assess the prostheses: the SHAP and the AHAP. As explained in the following sections, the first one is devoted to evaluating the performance of the device, and the second one focuses on the functional aspects of the hand. Although the four able-bodied subjects recruited were already familiarised with the use of the ABA, it was their first experience performing these kinds of tests. Therefore, each subject was allowed to practise for a short time before testing each prosthesis. In this way, the subject’s learning curve is placed on a plateau. For each subject, a minimum gap of two weeks was set between testing one prosthesis and the next to minimise the learning effect and prevent fatigue effects.

#### 2.3.1. Kapandji Opposition Test for Prosthetic Devices

**Figure 6:**
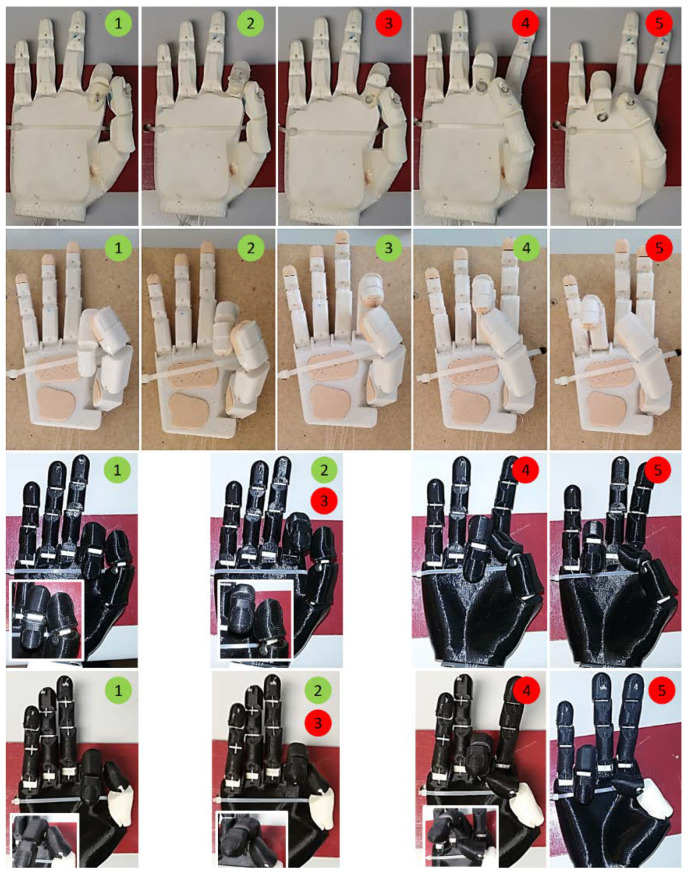
Results for the Kapandji test of the hands in the present research: from top to bottom, Dextrus, IMMA, Lb-45, and Lb-0 (green indicating success, red fail). Points 0 and 6 are not shown since no hand achieved them.

Figure 6 shows the Kapandji opposition test for the prosthetic hands studied here. It should be noted that only the IMMA hand reached stage 4 of the test. Dextrus was close to reaching stages 3 and 4, but excessive IP flexion prevented the hand from scoring. Both Limbitless hands reached stage 2 and failed at reaching stage 3. In the case of the Lb-45, it was not the orientation but the location of the thumb that made it contact the radial side index finger loosely in stages 1 and 2. The opposition of the thumb at Lb-0 made clear that it was not possible to reach stage 3, while stages 1 and 2 were achieved naturally.

#### 2.3.2. Reduced Southampton Hand Assessment Procedure (SHAP) 

The SHAP is a clinically validated hand function test developed by Light et al. [54,55] to assess the effectiveness of upper limb prostheses. The SHAP is made up of 6 abstract objects (in two versions, made of light and heavy materials) and 14 activities of daily living (ADL). Each of the 26 tasks focuses on one of the six main grasp types (GTs) of the human hand (spherical, power, tip, tripod, lateral, and extension). The subject sits at a table, and the objects are placed on the board provided within the SHAP set. Each task is timed by the participant and documented on an assessment sheet by the assessor. Subsequently, the recorded times can be normalised to 100, with greater scores denoting outstanding performances. This process provides six Functionality Profiles (quantifiable assessments of hand function for the six GTs) and an Index of Function (an overall assessment of hand function). A profuse description of the original procedure can be found in [29].

**Figure 7:**
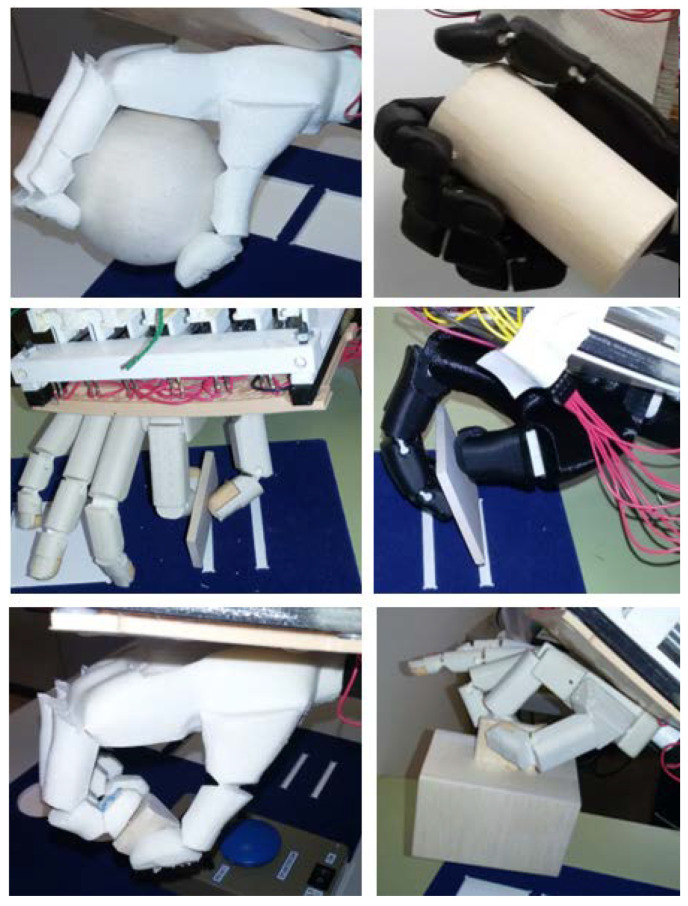
Tested hands performing SHAP’s LAO tasks. The six objects of the LAO set are shown.

In this study, a reduced version of the SHAP was used, as justified by the same authors in [35]. It requires only a part of the original SHAP to assess the prosthetic hands, that is to say: the one with six light abstract objects (LAO), see Figure 7. The protocol follows the SHAP manual itself. However, circumventing the original proposal gives a clearer insight into the GTs that the hand can perform. The abstract objects are shaped to encourage the use of the above-mentioned six standard GTs of the human hand, and they are named accordingly to that same prehensile pattern, e.g., Lightweight Lateral (Lateral L, for the sake of brevity). LAO tasks also have the advantage of focusing on prehensile ability: as they involve short transports, the influence of gross upper-limb movements is minimal. This testing might give some insight into the ability to take, or not, the grasping taxonomies that give their name to the respective LAO task. In reality, depending on the design of the prosthetic hand, some of these prehensile patterns cannot be truly achieved. However, as in the original SHAP, only the task completion time was taken into account. It is important to remark that SHAP focuses primarily on the above-mentioned performance. 

Three able-bodied subjects participated in this experiment. The order of the devices as they were fastened to the ABA to be tested consecutively by the three subjects was: Lb-45, Dextrus, Lb-0, and IMMA. According to the SHAP manual, subjects sat at a table with their arms resting on the table and elbows at almost 90° angle, and the SHAP kit was placed right in front. Task instructions were given before each task with the LAO. The tasks were self-timed: the subject started each task seated, with the prosthetic hand open, and pressed a chronometer before and after each task to record the time it took. 

The scoring system used adapts the Linear Index Function (LIF) [56] to the LAO set, with proven equivalent results to the SHAP’s original system [35], namely:

(1) $$LIF_{LAO}=\frac{1}{6}\cdot \overset{6}{\underset{l=1}{\sum }}T_{s_{l}}$$

In the expression (1), $T_{sl}$ is the Transformed Time Score registered for each of the SHAP tasks with LAO achieved within t seconds, and it is calculated as a percentage of mastery which considers a task limit time value of eight times the normative mean time (n) documented for the healthy hand [54,56]:

(2) $$T_{s}=\frac{8\cdot n-t}{7\cdot n}\cdot 100$$

$T_{s}$ is zero if the invested time for the task is greater than the referred as limiting time for that task, and 100, if the time invested is exactly n [56]. $T_{s}$ over 100 would mean outstanding performances.

*LIF_LAO_* evaluates the hand function relative to undamaged persons by measuring the time-to-accomplishment of the tasks. The nominal score test is 100 (*LIF_LAO_* of a typical healthy human hand over all the tasks), with lesser scores indicating a degree of impairment and greater scores indicating exceptional performance. 

#### 2.3.3. Anthropomorphic Hand Assessment Protocol (AHAP)

The AHAP [36] was developed by our group to address the need for benchmarking in grasping research. The AHAP uses 25 objects from the Yale-CMU-Berkeley (YCB) Object and Model Set, thereby enabling replicability. Across 26 postures/tasks, this protocol considers two additional GTs to those of the SHAP up to a total of eight, namely: the diagonal volar grip (DVG) and the hook grip (H) apart from the above-mentioned spherical grip (SG), power or cylindrical grip (CG), tip or pulp pinch (PP), tripod pinch (TP), lateral pinch (LP), and extension grip (EG). Additionally, it evaluates two non-grasping postures (platform (P) and index pointing/pressing (InP)). 

With each of the 25 objects, and after one minute of practice with each one, the subject performs a two-step task. In the first step (grasping), the operator hands the object over to the subject in the appropriate position to ease the correct execution of the GT. Once the prosthetic hand has made the grip, the operator releases the object, and the prosthetic device should hold the object for three seconds with the palm facing upwards. For the second step (maintaining), while preserving the grip, the subject slowly rotates the hand for the palm to face downwards and keeps the grip for three additional seconds. Each task with each object is repeated three times.

The AHAP allows inferring the degree of anthropomorphism in artificial hands by evaluating its functionality through a numerical Grasping Ability Score (GAS). The GAS involves the ability to replicate the human-like GTs (i.e., the above-mentioned function) and the effectiveness for maintaining these grasps under motion (in a sort of behaviour evaluation). It should be noted that the subject is not grasping the object on its own from a table as in evaluating the performance with the SHAP. All in all, the maximum GAS that an anthropomorphic artificial hand could achieve (100%) corresponds to the healthy human hand. The minimum GAS of 0% describes an artificial hand unable to grasp any object. In addition, AHAP provides an analysis for each GT through the partial GAS (pGAS). 

One able-bodied subject participated in the experiment using the ABA, as a previous study [42] demonstrated that the effect of the subject on the GAS was non-significant. The order of the devices as they were fastened to the ABA to be tested was: IMMA, Lb-45, Dextrus, and Lb-0. 

## 3. Results

### 3.1. SHAP Results

**Figure 8:**
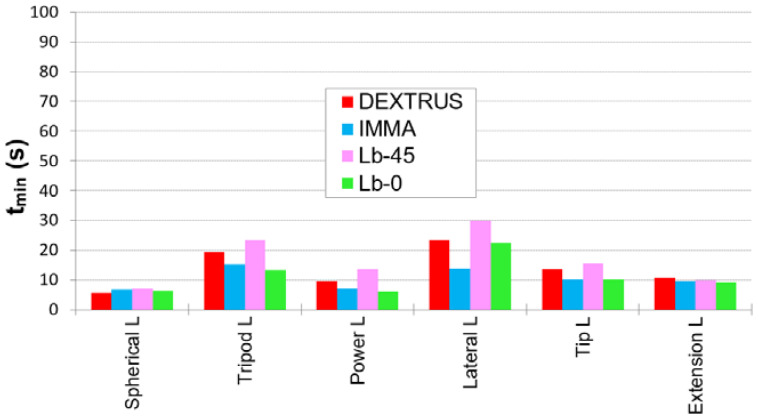
Fastest time (*t_min_*) ever registered for each of the LAO tasks.

Figure 7 shows some examples of the various hands performing some of the SHAP tasks with the LAO. Figure 8 shows, for each prosthesis, the fastest times (*t_min_*) in performing the tasks with each of the objects of the LAO set out of all the trials performed by the three subjects. The *t_min_* observed for each of the tasks across the three subjects highlights the best performances achieved under the boundary conditions of this research and may give an insight into the performance of each prosthesis in the daily life of a well-trained user. In other words, they give us the perspective of the usefulness of these hands from the best-case scenario. All the tasks with LAO were successfully completed at least once by one of the three subjects and in less than 30 s. The Lateral L task was the most difficult because the subject had to grasp the object’s handle (see Figure 7) away from the object’s centre of mass. As a result, the object was transported tilted and unsteady. Additional observations from the test were: for the Tripod L with the Lb-45 hand, the piece was subtly picked, and the final fit into the board slot was done by dragging the object; for the Power L with Lb-0, due to the little opposition of the thumb, the power grip was not possible against the diameter of the SHAP cylinder and the cylinder was instinctively approached from the top; for the Tip L and Extension L, with either Lb-0 and Dextrus, the grasp was achieved against the dorsum of the thumb, the latter being completely flexed, and it was also the solution adopted by two of the three users with the IMMA hand. The mean time for the tasks with LAO, averaged over the three subjects, was shortest for the IMMA (10.42 s), followed by the Lb-0 (11.25 s), Dextrus (13.71 s), and Lb-45 (16.49 s).

**Figure 9:**
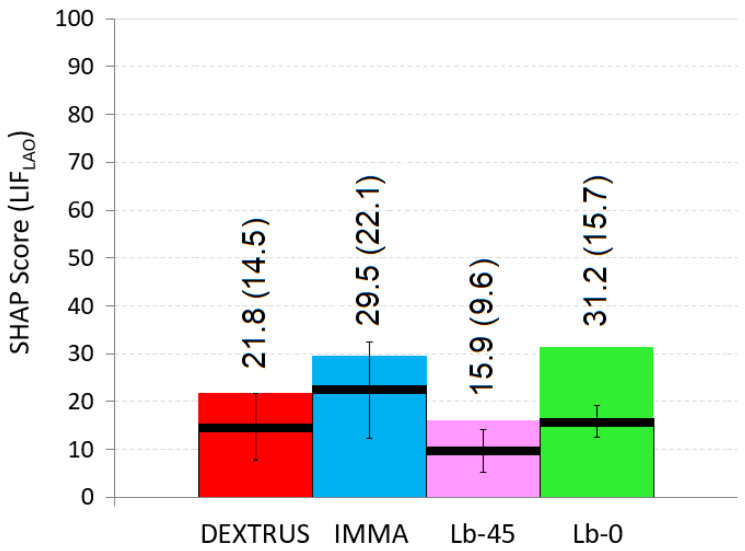
For each hand, the coloured bars indicate the *LIF_LAO_* scores for the fastest time ever registered across the three subjects. The black lines represent the mean value (value in brackets) and standard deviation amongst the three users.

In Figure 9, and for each hand, the coloured bars indicate the *LIF_LAO_* scores calculated with expression (1) taking into account, for each of the six LAO tasks, the fastest time ever (*t_min_*) registered across the three subjects (i.e., considering the best-case scenario of Figure 8). In the same Figure 9, and also for each hand, the black lines represent the mean value (value in brackets) and standard deviation of the *LIF_LAO_* scores for all three users (i.e., considering the *t* observed throughout each task, and after obtaining the LIFLAO for each one of the three subjects). Under the conditions of the present research, the Lb-0 obtained the highest score ($LIF_{LAO}$ = 31.2), closely followed by the IMMA ($LIF_{LAO}$ = 29.5). It should be noted that, in general, they both registered the shortest *t_min_* with each of the LAO objects, as depicted in Figure 8. On the other side, the Limbitless-45 obtained the worst *LIF_LAO_* scores, in agreement with having the longest *t_min_* with LAO.

Table 2 shows the $T_{s}$ calculated as per expression (2) with the shortest time registered for each hand in each task across the three subjects. By evaluating each $T_{s}$ we get more insight into the GTs, which each hand performed better. 

### 3.2. AHAP Results

Table 3 shows the results of the AHAP for each prosthetic hand performing the various GTs of this protocol. Those GTs considered in the AHAP, which were also considered in the SHAP, have been written in bold, with the name that the SHAP protocol uses in brackets. The overall *GAS* scores are listed at the bottom of the table.

IMMA shows the best GAS, although this scoring is similar for the rest of the hands. It should be noted that all these affordable hands got similar Maintaining scores, and it was the Grasping abilities that made the difference. Regarding the GTs, IMMA had distinguished performances except for the EG and the PP, where Lb-45 and Lb-0 scored better, respectively. Lb-0 and Lb-45 failed at P grasp due to the lack of compliance in the absence of the CMC joint. 

## 4. Discussion

Global AHAP and SHAP scores (*GAS* and *LIF_LAO_*, respectively) are consistent, as they show IMMA and Lb-0 as the best options from a global point of view. Furthermore, the AHAP protocol allows us to discern the proficiency of these hands into two additional interpretations: Grasping and Maintaining (in a sort of function and behaviour, as exposed in the introduction). The IMMA hand got the best score at Grasping by far. Grasping scores for the rest of the hands were similar, with Lb-45 four points ahead of the Dextrus and Lb-0. However, Lb-0 scored slightly better than the rest of the hands at Maintaining. Dextrus was last at both Grasping and Maintaining scorings. IMMA and Dextrus, with 3 DOF at the thumb, ranked first and last at GAS, respectively. IMMA also led the *LIF_LAO_* from the SHAP. The *LIF_LAO_* of the Dextrus was closer to the bottom of that ranking, between both Lb-0 and Lb-45 models. It may demonstrate the utility of having one specific DOC for one DOF of Ab/Ad at the CMC joint of the thumb, decoupled from the other DOC that actuates the thumb F/E. The only drawback is that it makes the control more cumbersome because of the additional DOC, yet the findings suggest it is worthwhile. 

**Figure 10:**
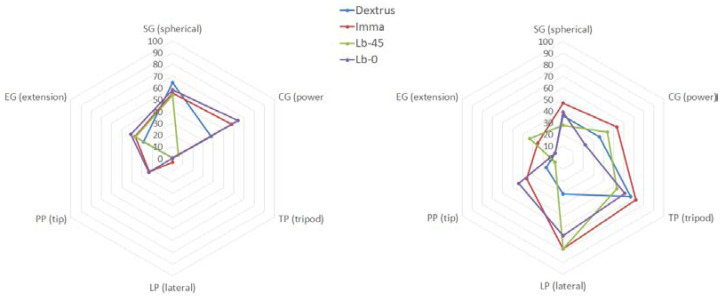
Left, *Ts* scores across the various prehensile patterns of the SHAP; right, AHAP’s *pGAS* for those same patterns.

Leaving aside the fact that *LIF_LAO_* and AHAP’s *GAS* offered like-minded global results, it is interesting to observe and interpret the evaluation for each of these GTs. For clarity, both denominations of the six GTs that SHAP and AHAP contemplate are shown in Figure 10. It is important to note that the opposition of the thumb has a great influence on all these GTs. It can be appreciated how IMMA (which reached stage 4 in the Kapandji opposition test) and Lb-0 (which reached stage 2 more convincingly than the other two hands) have two of the widest outlines.

**Figure 11:**
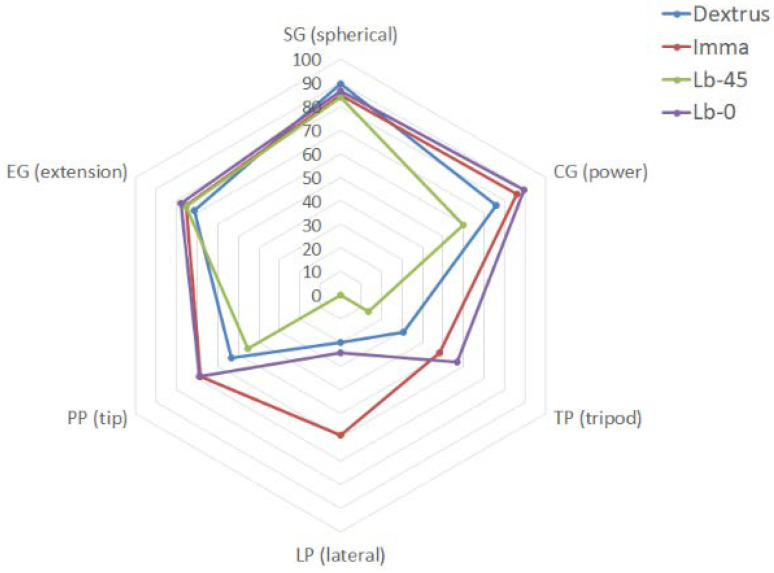
*Ts* scores across the various prehensile patterns of the SHAP considering the mean and limit times doubled concerning those of [54].

As stated earlier, SHAP has been submitted to some debate [35,56] and, afterwards, the linear scoring system and the use of the LAO set seem to provide a clearer insight into the capabilities of any prosthetic hand for the most basic performance expected. Hands confronted with basic shapes representative of most common handling situations in ADL result in more obvious interpretations. Such is the case of the spherical and power grasps: all hands performed quite well in front of these two objects, which are the biggest of the LAO set. Extension and tip grasps are two other basic grasps to aim at, and the scores dropped for all hands as the tiny plates used are more challenging. Together with the poor results obtained with the tripod grasp, it confirms that the use of these assistive devices is more suitable for bigger objects. Finally, the inherent difficulty in grasping the lateral object of the LAO set gives rise to a debate. Troubles come from its mass distribution and, again, one can ponder on the need for this grasp in the context of assistive devices where the solution towards performance also comes with the use of adapted objects [33]. That said, it should be noted that the current linear scoring system observes the reference values given for the healthy hand at [54], that is, the mean time and time limit shown in Table 2. It should be noted that we want to evaluate assistive devices, and not the rehabilitation of human hands, as originally the SHAP was intended for. In the context of prosthetic hands, it may make the evaluation too sharp: mean and limit times should be reviewed to tune the comparative in terms of avoiding null scores and thus being able to better compare the hands amongst themselves. In this regard, Figure 11 shows the same comparison with the mean and limit times from Table 2 doubled.

Again, not only by observing Figure 10-right but also now Figure 11, it is clear the importance of having two independent DOCs at the thumb, one for F/E and one at the CMC for thumb Ab/Ad, as the IMMA hand plot almost encircles the rest of the plots in this comparison. On the other side, although the Lb-45 performed averagely in some grasps, its plot tends to cover the smallest area in both studies, pointing out the inconvenience of such a fixed degree of palmar abduction. Finally, Figure 11 shows that having a coupled DOF at the CMC, like in the Dextrus, is not necessarily better than removing this joint with an appropriate fixed orientation because the plot of Lb-0 encircles that of the Dextrus. 

That said, it is interesting to note the great differences observed between the two Limbitless hands in the two protocols regarding the tripod and lateral grasps. The greater disparity observed with the SHAP (see Figure 11) may be due to the specificity of the tripod and lateral objects used. Both objects are quite difficult to grasp from the table, while they are offered to the hand and taken properly in the AHAP protocol (see Figure 10-right). That said, the Kapandji test already showed that Lb-0 had a better opposition aiming for these tasks, as the contact of the thumb with the index finger at Lb-45 was feeble (see Figure 6).

**Figure 12:**
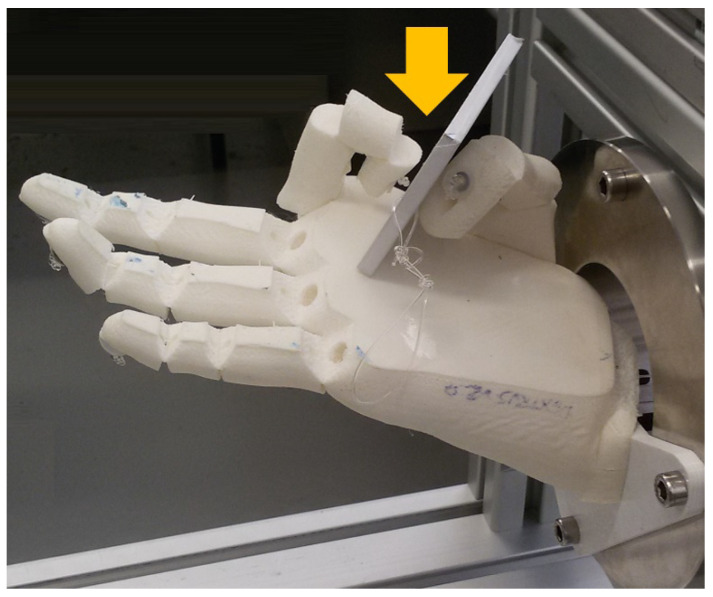
Under high tension at the tendons, Dextrus digits bend excessively, and the hand cannot grasp thin plates with LP, PP or EG (see gap pointed out, for PP). Although some functionality was demonstrated, this hand had to perform the SHAP tasks with the plate using alternative ways of grasping.

Some discussion may arise for the whole set of hands by observing Figure 10-right. The pGAS scoring is more analytical than the SHAP in evaluating the grasp taxonomies, as it observes the proper execution of the GT at first (i.e., function) and then the ability to hold the object against gravity (behaviour). Such a precise observation is not requested within the SHAP, being the shape of the object that hints at performing a GT. Forcing a hand to grasp an object in a precise specified manner, the object being offered by the operator, could appear to be counterproductive for knowing the actual final performance, that is, for self-achieving a task as observed with the SHAP. Nevertheless, in the context of the present research, this distinction is very interesting to get insight into the pros and cons of the different orientations of the thumb, namely:

- SG (spherical): Although all hands performed SHAP quite well, a fixed palmar abduction of the thumb may cause instability in grasping the different sizes of the spherical objects used at the AHAP.
- CG (power): again, high variability in the pGAS for the Limbitless models is observed due to the dependence between the abduction of the thumb and the various objects of the AHAP. It should be noted that Lb-0 got the best Ts with the SHAP for Cylinder L, but the object was instinctively grasped from the top. Again, it gives rise to a debate about getting a good performance while the functionality of the cylindrical grasp in this model was scarce.
- TP (tripod): being one of the trickiest GT due to the need for coordination amongst the thumb, the index and medium fingers, all hands showed having this function (all have similar pGAS), with IMMA and Dextrus taking the best scores. Leaving aside the fact that the Lb-0 had the proper abduction of the thumb for this particular SHAP task, the comparison between the performances of the IMMA and Dextrus backs up the convenience of having two independent DOCs at the thumb for F/E and Ab/Ad.
- LP (lateral), PP (tip), and EG (extension): these three grasps at the SHAP involve grasping a fine plate of balsa wood. The first observation is that Dextrus does not function properly for these GTs (see Figure 12). Accordingly, note that functionality was merely demonstrated in the Kapandji (see Figure 6) and the pGAS (Figure 10-right, using some thicker objects of the YCB set). That said, the performance is a dissimilar approach when assessing the hand with the SHAP. The rated performance results from instinctive alternatives of grasping, but all of them are highly unstable., such as pressing against the dorsum of the thumb, as mentioned in Section 3.1. Regarding the Limbitless models, while they both provide the function of LP (as seen with the Kapandji test), they swap their places in the ranking when it comes to the PP and EG (see Figure 10-right). It also may be due to what was pointed out with the Kapandji test, that is, how the force is exerted: Lb-0 may exert a greater closure force against the index alone for a sort of PP grip; Lb-45 has a loose contact with the radial side of the index finger but opposes better against the whole set of fingers for an EG.

The scope of the present research focused on affordable prosthetic devices, mainly devoted to amputees in low-resource sceneries. In the short term, the main goal of a designer should be to obtain functional prostheses that make life easier for users, with basic needs as the first to be covered. Their usefulness turns out to be subjective and multifactorial, i.e., it depends on various factors that may compensate for each other without it being clear their relevance in each particular situation. This poses the need to be pragmatic when considering the functionality intended vs the performance obtained by the user. The ultimate interest is to discern practical information for the designers regarding the design of the CMC joint in these kinds of affordable devices. SHAP only with LAO showed great sensitivity to the effects of the thumb design on their performance. At this point, it is important to note the coexistence of different assessment procedures that give complementary information: the tests for evaluating the performance should consider focusing on the success or failure of the tasks to be performed, while tests for evaluating the functionality do have to consider the GT adopted and its basic behaviour. AHAP showed to be a useful set for this second purpose.

## 5. Conclusions

It is clear that, nowadays, the design of a human-like prosthetic hand is utopian in the context of affordable designs. A trade-off between grasp capabilities and having as many DOF as the human thumb has to be found. This study confirms that the current mechanical designs of the CMC seem fortuitous in that context.

The comparison of affordable prostheses points out the shortcomings of some designs. As observed with the two versions of Limbitless, thumb placement was critical to performance as it influenced the manipulability with objects of different sizes. The lack of the CMC joint in the thumb prevented its circumduction rotation. In the human hand, it is the movement required to alternate between an LP (lateral), and a CG (power) or PP (tip) grasps. It can be thought that the addition of a CMC joint for thumb circumduction would guarantee a better polyvalence for different object sizes, yet results demonstrate that it should be supplemented with one additional DOC for this additional DOF. 

In conclusion, the advantage of having two decoupled DOC at the thumb is not only evident in the functionality evaluated first with the Kapandji and then with the AHAP but also in the performance demonstrated in the SHAP tasks with LAO. The results of the IMMA hand assert this fact: one DOC moves the Ab/Ad at the CMC joint, and the other flexes the IP and MCP joint. The final design of the thumb is a tradeoff between desired versatility and control complexity, yet this additional DOC could be justified in this case. On the other side, if having an additional DOC is out of the debate, the results with both protocols reinforce the idea of avoiding underactuated F/E and Ab/Ad, like in the Dextrus, in favour of the thumb having a fixed palmar abduction. That said, excessive palmar abduction should be avoided, as justified by the better results of Lb-0 compared to Lb-45.

## Figures and Tables

**Table 1:** Main design characteristics of the selected affordable hands used for the present study.

Hand	Dextrus	Lb-0, & Lb-45	IMMA
Kinematic architecture	Underactuated (15 DoF > 5 DoC)	Underactuated (14 DoF > 5 DoC)	Underactuated (15 DoF > 6 DoC)
- Number of joints *	3f, 3th	3f, 2th	3f, 3th
- Long finger joints	DIP, PIP, MCP	DIP, PIP, MCP	DIP, PIP, MCP
- Thumb joints	IP, MCP, CMC	IP, MCP	IP, MCP, CMC
Materials (% infill)	Ninjaflex^®^ (35%)	PLA (25%)/Ninjaflex^®^ (joints, 25%)	PLA SOFT-Flexible (body palm, phalanges)/Ninjaflex^®^ (joints, 25%)/FilaFlex^®^ (finger pulps, inner palm)
Overall size (HB/HL, mm)	87/185	89/200	80/184.4
Weight (g) w/o actuators	131	144.5	131.5
License	CC BY-SA 4.0	CC BY-NC 3.0	CC BY-SA 4.0
Printing time	28 h	16 h	45 h
Material cost	$11	$6	$10

(*) 3f: three joints at fingers; 2f: two joints at fingers; 3th: three joints at the thumb; 2th: two joints at the thumb.

**Table 2:** Transformed Time Scores for each LAO task, calculated from the *t_min_* registered.

SHAP Task	SHAP Normative Data [54]		Dextrus		IMMA		Lb-45		Lb-0	
	Mean Time (s)	Time Limit (s)	*t_min_*	*T_s_*	*t_min_*	*T_s_*	*t_min_*	*T_s_*	*t_min_*	*T_s_*
Light sphere	*1.63*	*13.04*	5.68	64.50	6.72	55.39	6.94	53.46	6.40	58.19
Light tripod	*1.66*	*13.28*	19.40	0	15.31	0	23.47	0	13.42	0
Light power	*1.77*	*14.16*	9.50	37.61	7.00	57.79	13.47	5.57	6.19	64.33
Light lateral	*1.77*	*14.16*	23.37	0	13.72	3.55	29.94	0	22.31	0
Light tip	*1.59*	*12.72*	13.60	0	10.16	23.00	15.37	0	10.10	23.54
Light extension	*1.78*	*14.24*	10.69	28.49	9.63	37.00	9.72	36.28	9.10	41.25

**Table 3:** AHAP scores, pGAS, for each GT. The maximum pGAS score for each GT is in bold. Overall, AHAP scores are listed at the bottom.

%	Dextrus	IMMA	Lb-45	Lb-0
H	**83**	75	75	81
DVG	47	42	**58**	50
IP	**100**	83	**100**	**100**
P	**100**	**100**	0	0
SG (spherical)	36	**47**	28	39
TP (tripod)	67	**72**	53	61
CG (power)	36	**53**	44	22
LP (lateral)	31	**78**	**78**	67
PP (tip)	17	36	8	**44**
EG (extension)	8	25	**33**	8
Grasping	58	**78**	62	58
Maintaining	29	32	34	**37**
GAS	44	**56**	48	48

## Data Availability

Not applicable.

## Abbreviations

3D	Three-dimensional
Ab/Ad	Abduction/Adduction
ABA	Able-Bodied Adaptor
ABS	Acrylonitrile butadiene styrene
ADL	Activities of daily living
AHAP	Anthropomorphic Hand Assessment Protocol
BP	Body-powered
CAD	Computer Aided Design
CG	Cylindrical grip
CMC	Carpometacarpal
DIP	Distal interphalangeal
DOC	Degree(s) of control
DOF	Degree(s) of freedom
DVG	Diagonal volar grip
EG	Extension grip
F/E	Flexion/Extension
FDM	Fused deposition modelling
GAS	Grasping Ability Score
GTs	Grasp types
H	Hook grip
HB	Hand breadth
HL	Hand length
IP	Interphalangeal
InP	Index pointing/pressing
KC	Kinematic chain
L	Lightweight
LAO	Light abstract objects
Lb	Limbitless
LIF	Linear Index Function
LMICs	Low and medium-income countries
LP	Lateral pinch
MCP	Metacarpophalangeal
P	Platform
pGAS	partial GAS
PIP	Proximal interphalangeal
PLA	Polylactic acid
PP	Pulp pinch
ROM	Range of motion
SG	Spherical grip
SHAP	Southampton Hand Assessment Procedure
TMC	Trapeziometacarpal
TP	Tripod pinch
TPU	Thermoplastic polyurethane
YCB	Yale-CMU-Berkeley
